# MicroRNA Alterations in the Brain and Body Fluids of Humans and Animal Prion Disease Models: Current Status and Perspectives

**DOI:** 10.3389/fnagi.2018.00220

**Published:** 2018-07-23

**Authors:** Eirini Kanata, Katrin Thüne, Konstantinos Xanthopoulos, Isidre Ferrer, Dimitra Dafou, Inga Zerr, Theodoros Sklaviadis, Franc Llorens

**Affiliations:** ^1^Laboratory of Pharmacology, Prion Diseases Research Group, Department of Pharmacy, School of Health Sciences, Aristotle University of Thessaloniki, Thessaloniki, Greece; ^2^Department of Neurology, University Medical Center Göttingen, Göttingen, Germany; ^3^Laboratory of Pharmacology, Department of Pharmacy, School of Health Sciences, Aristotle University of Thessaloniki, Thessaloniki, Greece; ^4^Bellvitge University Hospital, Bellvitge Biomedical Research Institute, Barcelona, Spain; ^5^Department of Pathology and Experimental Therapeutics, University of Barcelona, Barcelona, Spain; ^6^Network Center for Biomedical Research of Neurodegenerative Diseases, Institute Carlos III, Ministry of Health, Madrid, Spain; ^7^Department of Genetics, Development, and Molecular Biology, School of Biology, Aristotle University of Thessaloniki, Thessaloniki, Greece

**Keywords:** microRNAs, prion diseases, sCJD, prion diseases animal models, brain, CSF, blood, neurodegenerative disorders

## Abstract

Prion diseases are transmissible progressive neurodegenerative conditions characterized by rapid neuronal loss accompanied by a heterogeneous neuropathology, including spongiform degeneration, gliosis and protein aggregation. The pathogenic mechanisms and the origins of prion diseases remain unclear on the molecular level. Even though neurodegenerative diseases, including prion diseases, represent distinct entities, their pathogenesis shares a number of features including disturbed protein homeostasis, an overload of protein clearance pathways, the aggregation of pathological altered proteins, and the dysfunction and/or loss of specific neuronal populations. Recently, direct links have been established between neurodegenerative diseases and miRNA dysregulated patterns. miRNAs are a class of small non-coding RNAs involved in the fundamental post-transcriptional regulation of gene expression. Studies of miRNA alterations in the brain and body fluids in human prion diseases provide important insights into potential miRNA-associated disease mechanisms and biomarker candidates. miRNA alterations in prion disease models represent a unique tool to investigate the cause-consequence relationships of miRNA dysregulation in prion disease pathology, and to evaluate the use of miRNAs in diagnosis as biomarkers. Here, we provide an overview of studies on miRNA alterations in human prion diseases and relevant disease models, in relation to pertinent studies on other neurodegenerative diseases.

## Introduction

Prion diseases are rapidly progressive, fatal neurodegenerative disorders, characterized by widespread neuronal loss, gliosis, spongiform change and deposition of the pathological PrP^Sc^ protein in the Central Nervous System (CNS) (Appleby et al., 2009). Similarly to other neurodegenerative disorders (NDs), dysfunctional proteostasis is a key feature in prion diseases. Changes in the cellular prion protein (PrP^C^) metabolism and its conversion into an aberrant isoform (PrP^Sc^) in a self-propagating manner are attributed to prion diseases pathogenesis (Colby and Prusiner, 2011). However, precise molecular pathogenic mechanisms remain unclear. Prion disease model systems, including animal and cell culture models, recapitulate different aspects of disease mechanisms and are valuable for exploring underlying pathogenesis (Zou and Gambetti, 2004; Watts and Prusiner, 2014; Brandner and Jaunmuktane, 2017).

miRNA dysregulation has been linked to several NDs (Hébert and De Strooper, 2009; Abe and Bonini, 2013; Tan et al., 2015), including prion diseases. miRNAs are a class of short, non-coding RNAs that regulate gene expression post-transcriptionally through translational inhibition and/or mRNA degradation. They are involved in several biological processes, including neuronal function and survival (Zhang et al., 2007). miRNA biogenesis includes several processing steps mediated by multiple miRNA maturating proteins in the nucleus and cytoplasm (Ha and Kim, 2014). miRNAs are usually transcribed by RNA polymerase II as long, primary miRNA transcripts and undergo a series of cleavage events by the ribonucleases Drosha and Dicer, as well as post-transcriptional modifications, such as splicing and editing (Slezak-Prochazka et al., 2010). Mature miRNAs interact with Argonaute proteins to form the miRNA-induced silencing complex (RISC), which mediates translational repression or target degradation through binding to mRNAs (Bartel, 2009; Hammond, 2015). Various regulatory mechanisms affect miRNAs processing efficiency and activity (Finnegan and Pasquinelli, 2013; Ha and Kim, 2014).

A collective view of miRNA alterations in prion disease patients and/or relevant disease models is currently unavailable. We review experimentally validated miRNA alterations in the brain and body fluids of human prion diseases and/or disease models. We compare these deregulated miRNAs with other NDs; we comment on possible outcomes of miRNA dysregulation in prion diseases and discuss potential underlying mechanisms of this deregulation. We further discuss the diagnostic and therapeutic potential of miRNAs. Finally, we present future perspectives in the prion diseases miRNA research field.

## miRNA Signatures in the Brain of Prion Diseases-Afflicted Individuals

Studies on miRNA alterations were conducted in the brain of patients afflicted with sporadic Creutzfeldt-Jakob disease (sCJD) (Montag et al., 2009; Lukiw et al., 2011; Llorens et al., 2018), Fatal Familial Insomnia (FFI) (Llorens et al., 2018) and Gerstmann–Sträussler–Scheinker syndrome (GSS) (Lukiw et al., 2011).

Altered miRNA expression has been investigated in animal and cell culture models including: (a) a sCJD mouse model that recapitulates CJD brain neuropathology (Padilla et al., 2011; Llorens et al., 2014, 2017, 2018), (b) BSE-infected macaques (Montag et al., 2009) (c) murine models intracerebrally inoculated with various scrapie strains [RML (Majer et al., 2012; Boese et al., 2016), 139A (Gao et al., 2016), Me7 (Gao et al., 2016) and 22A (Saba et al., 2008)] or scrapie infected SMB-S15 cells (Gao et al., 2016), (d) murine hypothalamic GT1-7 cells infected with a mouse adapted human GSS strain (Bellingham et al., 2012; Bellingham and Hill, 2017) and (e) murine neuroblastoma cells propagating the 22L scrapie strain (Montag et al., 2012).

We detected limited overlap among the altered miRNAs reported by these studies. This is likely explained by the multitude of factors that vary between analyzed prion models and/or strains, different miRNA profiling platforms and heterogeneity of analyzed tissues (bulk tissue, microdissected neurons, isolated synaptoneurosomes, etc.). When comparing different miRNA profiling platforms, interplatform reproducibility is often not fully achieved and even variations within the same platform have been described (Chugh and Dittmer, 2013). Methodological advantages, drawbacks and features, possibly contributing to limited results overlap are briefly discussed below.

Microarray technology, quantitative real-time reverse transcription PCR (qRT-PCR) and/or Northern Blot analyses were used for targeted miRNA profiling. Microarrays enabled simultaneous quantification of several miRNAs at the expense of specificity, due to the homogeneous hybridization conditions used. Individual miRNA qRT-PCR assays, based on either stem-loop or locked nucleic acid-modified (LNA) primers, enhanced specificity and enabled differentiation of mature miRNAs from precursors (Pritchard et al., 2012; Chugh and Dittmer, 2013). Even though highly sensitive, Northern Blot analysis, is time-consuming and laborious for large scale miRNA analysis (Koshiol et al., 2010).

Application of small RNA sequencing (RNAseq) allowed high-throughput analysis on single-nucleotide resolution, offering comprehensive and unbiased miRNA quantifications. However, method-dependent distortions in miRNA quantification have been reported. Multiple factors, such as RNA G/C-content or secondary RNA structures, can influence cDNA synthesis (Raabe et al., 2014). Overall, platform-specific biases affect the consistency and accuracy of miRNA profiling, which may contribute to variability in miRNA quantification (Chugh and Dittmer, 2013).

Despite the limited overlap, some miRNAs presented similar deregulation in at least two independent disease contexts. **Table 1** summarizes experimentally validated miRNAs found to display similar deregulation patterns in prion affected brain tissue, at clinical disease.

**Table 1:** Dysregulated miRNAs in prion diseases.

	Human prion diseases			*In vivo* prion models						
				sCJD	BSE	scrapie				
miRNA	sCJD	FFI	GSS	sCJD-MM1-tg340 mice	BSE-macaques	139A-mice	Me7-mice	S15-mice	22A-mice	RML-mice
**miR-146a-5p**	↑a, b, d, e ([1]; [2])		↑e ([2])	↑f, g ([1])		↑h ([3]; [2])	↑h ([3])	↑h ([3])	↑j ([4])	↑k, l ([5]; [6])
**miR-26a-5p**	↑a, b ([1])			↑f, g ([1])	↑i ([7])					↓k ([5])
miR-195-5p	↑a, c ([1])	↑f, g ([1])		↑f ([1])						
**miR-342-3p**	↑a, c ([1];[7])			↑f, g ([1])	↑i ([7])				↑j ([4])	
miR-16-5p	↑a ([1])			↑f ([1])						
miR-29b-3p	↑a, c ([1])			↑f, g ([1])						
Let-7i-3p	↑a, b, d ([1])			↑g ([1])						
miR-378a-3p	↑b, d ([1])			↑g ([1])						
miR-449a	↑a, b, c, d ([1])			↑g ([1])						
miR-154-5p	↑a, c, d ([1])			↑f, g ([1])						
miR-341-3p						↑h ([3])	↑h ([3])	↑h ([3])		
miR-3470a						↑h ([3])	↑h ([3])	↑h ([3])		
miR-3473a						↑h ([3])	↑h ([3])	↑h ([3])		
miR-3473b						↑h ([3])	↑h ([3])	↑h ([3])		
miR-879-5p						↑h ([3])	↑h ([3])	↑h ([3])		
miR-200b-5p						↑h ([3])	↑h ([3])			
**miR-124-3p**	↓a, b ([1])			↓f, g ([1])						↓k ([5])
**miR-141-3p**						↓h ([3])	↓h ([3])	↓h ([3])		↓l ([6])
miR-182-5p						↓h ([3])	↓h ([3])	↓h ([3])		
miR-200a-3p						↓h ([3])	↓h ([3])	↓h ([3])		
miR-96-5p						↓h ([3])	↓h ([3])	↓h ([3])		
miR-200b-3p						↓h ([3])	↓h ([3])			

*The table lists dysregulated miRNAs at clinical disease stage that have been validated in more than one human prion disease and/or prion disease model. In bold are indicated miRNAs detected as deregulated in more than one study. ^a^MM1 Frontal cortex. ^b^MM1 cerebellum. ^c^VV2 frontal cortex. ^d^VV2 cerebellum. ^e^Temporal lobe. ^f^Frontal cortex. ^g^Cerebellum. ^h^Cortex. ^i^Basis pontis. ^j^Whole brain. ^k^CA1 hippocampus neurons. ^l^Synaptoneurosomes forebrain. [1]: Llorens et al. (2018). [2]: Lukiw et al. (2011). [3]: Gao et al. (2016). [4]: Saba et al. (2008). [5]: Majer et al. (2012). [6]: Boese et al. (2016). [7]: Montag et al. (2009).*

Upregulation of miR-146a-5p was demonstrated in six independent studies, including sCJD and GSS (Lukiw et al., 2011), sCJD and the corresponding mice model (Llorens et al., 2018), mice scrapie models [139A (Lukiw et al., 2011; Gao et al., 2016), Me7, S15 (Gao et al., 2016), 22A (Saba et al., 2008)], and CA1 (*Cornu Ammonis* region 1) neurons (Majer et al., 2012), as well as forebrain synaptoneuroso (Montag et al., 2009). Analogous deregulation, also extending to 22A-scrapie mice, was observed for miR-342-3p (Saba et al., 2008). Upregulation of miRNA-195-5p was reported in FFI, sCJD and in sCJD-mice (Llorens et al., 2018). Increased levels of miR-16-5p, miR-29b-3p, let-7i-3p, miR-378a-3p, miR-449a, and miR-154-5p were observed in sCJD patients and in the sCJD mice model brain, suggesting disease-specific miRNA profiles (Llorens et al., 2018). Further, upregulation of miR-341-3p, miR-3470a, miR-3473a, miR-879-5p, and miR-200b-5p in the cortex of mice infected with several scrapie strains (Gao et al., 2016) suggests common responses at the miRNA level elicited by different scrapie strains.

Among downregulated miRNAs, miR-124-3p was reduced in the frontal cortex and cerebellum of both sCJD patients and sCJD mice (Llorens et al., 2018) and in RML-infected mice CA1 neurons (Majer et al., 2012). Other miRNAs downregulated in the cortex of mice infected with different scrapie strains were miR-182-5p, miR-200a-3p, miR-96-5p, and miR-200b-3p (Gao et al., 2016). A similar pattern was observed for miRNA-141-3p, which was also downregulated in RML-mice forebrain synaptoneurosomes (Boese et al., 2016).

### Temporal miRNA Expression Analyses in Prion Diseases

Temporal miRNA analyses in: (a) RML mice CA1 microdissected neurons (Majer et al., 2012; Burak et al., 2018), (b) RML mice forebrain and hippocampus synaptoneurosomes (Boese et al., 2016) and (c) sCJD mice cortex and cerebellum (Llorens et al., 2018), suggest distinct and dynamic miRNA signatures in disease progression. A trend toward upregulation for miR-124-3p, miR-16-5p, miR-26a-5p, miR-29a-3p, miR-132-3p, and miR-140-5p at pre-clinical stages, followed by downregulation to basal levels during disease progression and a further reduction throughout the clinical disease stage was identified in RML mice CA1 neurons; miR-146a-5p displayed increased levels throughout disease progression, reaching its highest levels at preclinical stages (Majer et al., 2012). Upregulation of miR-16-5p was also observed during early prion disease in RML mice neurons by a follow-up study (Burak et al., 2018). Upregulation of miR-124-3p was also detected in pre-clinical RML mice synaptoneurosomes, along with miR-32-5p, miR-136-5p, miR-150-5p, miR-345-5p, and miR-361-5p, while miR-141-3p, miR-183-5p, and miR-200c-3p were reduced in the same preparations (Boese et al., 2016).

Interestingly, among a pool of miRNAs specifically dysregulated in clinical sCJD disease, miR-124-3p and miR-16-5p change their expression during early symptomatic phase in sCJD mice (Llorens et al., 2018), suggesting their contribution in pre- and early clinical disease mechanisms.

**FIGURE 1:**
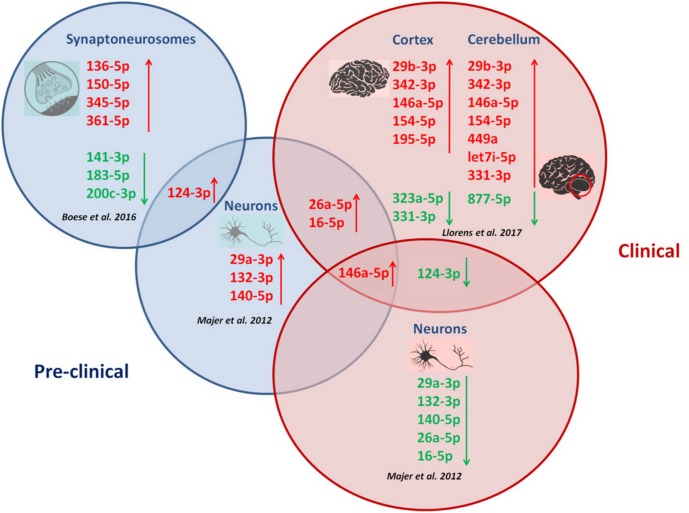
Correlation between miRNA alterations and disease progression in prion diseases. Temporal miRNA profilng during prion disease progression in CA1 neurons (Majer et al., 2012), synaptoneurosomes (Boese et al., 2016) and brain regions (Llorens et al., 2017) revealed distinct miRNA alterations with disease progression. Different disease stages are featured by specific miRNA signatures. Upregulation of miR-146a-5p, miR-16-5p and miR-26a-5p appeared in two prion disease models across disease progression. Downregulation of miR-124-3p was specifically observed during clinical prion disease stages, while the same miRNA (miR-124-3p) was found upregulated in CA1 neurons and synaptoneurosomes at pre-clinical stages in two prion disease models.

A schematic illustration of temporal miRNA alterations during disease progression is shown in **Figure 1**.

### miRNA Deregulation Patterns in Prion Diseases and Other NDs

Some miRNAs dysregulated in prion diseases are also altered in the brain of other NDs, displaying a similar or contrasting trend. The upregulated in prion diseases miR-146a-5p has been found overexpressed in active Multiple Sclerosis (MS) lesions (Junker et al., 2009), and in Alzheimer’s disease (AD) brain (Sethi and Lukiw, 2009; Müller et al., 2014) associated with different cellular contexts, including neurons (Wang et al., 2016) and astrocytes (Cui et al., 2010; Arena et al., 2017). Differential miR-146a-5p expression is suggested by *in vitro* studies in brain cells contributing to inflammatory cell type-specific functions during neurodegeneration (Li et al., 2011). Similar to miR-146a-5p, miR-195-5p is upregulated in prion disease brain and in inactive MS lesions (Junker et al., 2009); miR-16-5p is upregulated in both prion diseases and AD brain (Müller et al., 2014) and miR-26a-5p is increased in prion diseases and in dopaminergic midbrain Parkinson’s disease (PD) neurons (Briggs et al., 2015).

On the contrary, miR-29b-3p displays opposite regulation in MS and AD compared to prion diseases, as it is downregulated in normal appearing white matter (NAWM) (Noorbakhsh et al., 2011) and in chronic MS lesions (Lau et al., 2013); the same miRNA is decreased in the anterior temporal cortex and cerebellum of AD patients with increased BACE1 expression (Hebert et al., 2008).

Similar miRNA alterations in prion diseases and other NDs may indicate common miRNA-regulated molecular pathways. Identification of prion-disease-specific miRNA alterations in human brain, followed by robust confirmation in appropriate models, are required to gain insights into the specific role of miRNAs deregulation in prion diseases, and/or therapeutic potential.

## Functional Implications of miRNA Alterations in Prion Diseases

One outstanding question is to what extent miRNA alterations (**Table 1**) reflect prion disease processes. Since miRNAs are involved in the regulation of complex gene networks, reported miRNA alterations in prion disease have the potential to be involved in virtually every aspect of disease pathophysiological mechanisms.

Interesting functional implications for potentially disease-associated miRNAs have been observed. The brain-enriched, NF-κB-sensitive miR-146a-5p has been suggested as a mediator of inflammatory microglial responses in prion disease (Lukiw et al., 2011; Saba et al., 2012). Additionally, compelling evidences revealed significant miR-146a-5p overexpression in prion disease neurons (Majer et al., 2012; Boese et al., 2016; Llorens et al., 2018), suggesting that neuronal miR-146a-5p induction is triggered by pathological stimuli. Further, miR-146a-5p overexpression in a neuronal cell culture model resulted in MAP1B downregulation, indicating miRNA-mediated mechanisms involved in microtubules networks regulation, with extensions to synaptic plasticity (Chen and Shen, 2013). Moreover, miR-146a-5p has been linked with regulation of reactive oxygen species generation (redoximiR), through NOX4 repression (Cheng et al., 2013).

The neuron-enriched miR-124-3p has been associated with neurite outgrowth (Maiorano and Mallamaci, 2009), dendritic complexity (Sanuki et al., 2011) and cholinergic anti-inflammatory responses (Sun et al., 2013), suggesting that its pre-clinical upregulation may reflect compensating mechanisms to overcome synaptic dysfunction (Cunningham et al., 2003; Hilton et al., 2013) and inflammatory processes (Carroll et al., 2015) during early disease responses.

*In vitro* studies revealed that lentiviral-based miR-16-5p overexpression, mimicking miR-16-5p upregulation observed in disease condition, induce reduced neurite length and branching of cultured hippocampal neurons (Burak et al., 2018). Also neuroprotective roles were suggested for miR-16-5p in the aging brain (Parsi et al., 2015), indicating multivariable effects of single miRNAs on cellular processes (Wilczynska and Bushell, 2015).

All the above highlight the complexity of miRNA-mediated regulation of cellular processes, stressing the need for further studies to globally identify miRNA-mRNA interactions within the disease context.

## Mechanisms of Altered miRNA Signatures in Human Prion Diseases

Expression and cellular miRNA abundance is influenced by mechanisms including genetic and epigenetic factors, RNA editing, alteration of transcription factors targeting miRNA, altered miRNA biogenesis, miRNA turnover and/or miRNA sorting in specific cellular compartments (Schanen and Li, 2011; Bronevetsky and Ansel, 2013; Tomaselli et al., 2013; Gulyaeva and Kushlinskiy, 2016).

Genetic variations, such as single nucleotide polymorphisms (SNPs) in miRNA genes or their target sites affecting miRNA expression, biogenesis and target binding (Jin and Lee, 2013; Saba and Booth, 2013) have been associated with NDs (Saba et al., 2014; Zhang et al., 2015; Moszyńska et al., 2017). A miR-146a promoter SNP (rs57095329) is associated with susceptibility to FFI and correlated with appearance of mutism and detection of the 14-3-3 protein in the Cerebrospinal Fluid (CSF) of sCJD patients (Gao et al., 2017). The same SNP has been suggested as a risk factor for AD (Cui et al., 2014), while another SNP (rs2910164) residing in pri-miR-146a, has been associated with AD (Zhang et al., 2015) and MS (Kiselev et al., 2015; Li et al., 2015; Park et al., 2016; Zhou et al., 2017).

Dysfunctional regulatory pathways involving transcription factors may underlie miRNA alterations in prion diseases. A pool of miRNAs upregulated in sCJD brain has been observed to be under the control of STAT3 (Llorens et al., 2018), which is upregulated and activated in prion diseased brain (Llorens et al., 2014; Carroll et al., 2015), suggesting a STAT3-dependent mechanism of miRNA deregulation in sCJD.

**FIGURE 2:**
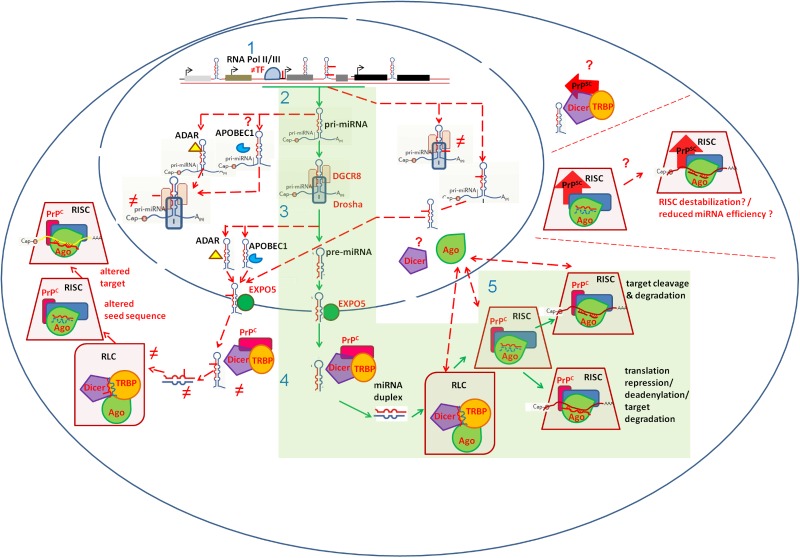
Schematic representation of cellular mechanisms possibly implicated in miRNA dysregulation in prion diseases. The canonical miRNA biogenesis and function pathway is indicated in green boxes. Mechanisms possibly contributing to miRNA dyregulation in prion diseases are indicated with dashed red lines. miRNA genes, residing in exonic, intronic or intergenic regions, are transcribed by RNA polymerase II or III **(1)**. Altered miRNA gene transcription rate may impact miRNA levels. This can be associated to altered (≠) abundance of Transciption Factors (TF) inducing miRNA gene expression (e.g., STAT3), and/or to Single Nucleotide Variants (SNPs) residing within miRNA-gene promoter regions (e.g., rs57095329, illustrated as solid red line). Upon transcription, pri-miRNAs’ flanking sequences are removed by the microprossesor complex, containing Drosha and DGCR8 **(2)**. This processing generates pre-miRNA stem-loop structures **(3)**. Pre-miRNAs are exported into the cytoplasm through Exportin5 (EXPO5). Dicer removes the pre-miRNA loop, generating a miRNA duplex **(4)**. SNPs in miRNA genes may interfere with pri- and/or pre-miRNAs processing by Drosha/DGCR8 **(2)** and Dicer **(4)** respectively. RNA editing, mediated by ADARs and/or APOBEC1, may occur in pri- and/or pre-miRNAs, introducing Single Nucleotide Variants (SNVs) with the potential to affect miRNA maturation **(2, 4)**. Reduced Drosha, DGCR8 and Dicer levels, reported in human prion diseases, may impact miRNA maturation **(2, 4)**. Following Dicer processing, the miRNA duplex is loaded from the RISC-loading complex (RLC) onto RISC. Ago proteins, are core components of RISC, forming the active functional unit of the miRNA-mediated gene regulation **(5)**. Abnormal subcellular localization of Ago proteins, reported in sCJD, may affect miRNA machinery **(5)**. PrP^C^-Ago interactions, which stabilize RISC, may be affected by PrP^Sc^ presence. Similarly, PrP^Sc^ could possibly impact PrP^C^-Dicer interactions and thus miRNA maturation and function. Incorporated into the RISC complex, miRNAs promote either translational repression or mRNA degradation, according to sequence complementary **(5)**. SNPs in miRNA seed sequences and/or RNA editing events possibly occurring within miRNA seed sequences may impact miRNA-target mRNA interactions and even redirect miRNAs in new targets **(5)**.

Disruption of miRNA biogenesis may contribute to miRNA dysregulation in prion diseases. Reduced levels of the essential miRNA maturing proteins Drosha, its cofactor DGCR8 and Dicer have been reported in sCJD patients brain (Llorens et al., 2018). Argonaute proteins, essential components of the miRNA-guided gene regulation, show altered subcellular distribution in sCJD brain (Llorens et al., 2018). Interestingly, PrP^C^ has been shown to interact with Argonaute proteins under physiological conditions, promoting and stabilizing RISC complexes (Gibbings et al., 2012). Whether the presence of PrP^Sc^ or the lack of PrP^C^ functionality directly contributes to miRNA dysregulation in prion disease deserves investigation. **Figure 2** summarizes potential mechanisms of miRNA deregulation in prion diseases.

Other NDs also display deregulated miRNA biogenesis (Tan et al., 2015); upregulation of Drosha, DGCR8 and Dicer has been observed in MS patients brain (Jafari et al., 2015); further, abnormal cellular distribution of Drosha (Porta et al., 2015) and Dicer dysfunction (Emde et al., 2015) have been reported in ALS patients brains.

## Altered miRNAs as Potential Diagnostic Tools in Prion Diseases

Peripheral circulating miRNA profiles corresponding to miRNAs from peripheral blood mononuclear cells (PBMCs), blood plasma/serum and CSF, either as free-circulating molecules or as exosome contents, may provide important indicators of pathophysiological processes in the brain (Sheinerman and Umansky, 2013; Grasso et al., 2015).

In contrast to other NDs such as AD (Kiko et al., 2013; Müller et al., 2014; Lusardi et al., 2016) and MS (Quintana et al., 2017), very limited information on experimentally validated miRNA alterations in CSF from healthy and prion diseases affected individuals is available. A preliminary analysis including a small number of miRNAs selected based on their deregulation in sCJD human brain, indicated low correspondence between altered miRNAs in sCJD brain and CSF. Several miRNAs displaying increased levels in sCJD brain, such as miR-26a-5p, miR-195-5p, let-7i-3p, miR-378a-3p, miR-449a, miR-124-3p, and miR-154-5p, did not show significant differences in sCJD CSF samples compared to healthy individuals (Llorens et al., 2018). However, identification of miR-204-5p differential levels in the CSF of controls and sCJD patients, indicates the potential use of CSF miRNAs as biomarkers for prion diseases diagnosis.

Regarding the diagnostic potential of miRNAs in prion diseases utilizing less invasive sampling methods, a recent study reported increased levels of circulating miR-342-3p and miR-21-5p in the plasma of sheep naturally affected by scrapie (Rubio et al., 2017), encouraging high-throughput analyses of plasma miRNAs in animal and human prion disease cases in relation to control samples and further validation of the most potent targets.

Interestingly, increased levels of miR-342-3p and miR-21-5p were also reported in exosomes released from murine hypothalamic cells infected with the mouse-adapted M1000 strain of human GSS (GSS-GT1-7) (Bellingham et al., 2012). Exosomes are lipid vesicles derived from most cells, including neurons and glia; they carry specific enriched subsets of nucleic acids, including miRNAs and mRNAs, various proteins and lipids. Exosomal pathways are considered as contributors to inter-cellular communication, and to several neurodegenerative states by spreading pathological proteins and other disease-associated molecules (Hartmann et al., 2017; Soria et al., 2017). In prion diseases, the exosomal miRNA provides an appealing approach for disease-specific diagnostic signatures. Since exosomes may be detected in CSF, blood plasma or serum, it is tempting to speculate that exosomes isolated from body fluids of prion affected individuals would present specific miRNA patterns of potential diagnostic value. Toward this goal, miR-21-5p and miR-322-5p (also identified as upregulated in exosomes from GSS infected cells, Bellingham et al., 2012), not previously suggested as biomarkers in blood serum/plasma of other NDs such as AD (Geekiyanage et al., 2012; Kiko et al., 2013; Dong et al., 2015; Guo et al., 2017; Nagaraj et al., 2017; Zeng et al., 2017), MS (Vistbakka et al., 2017), PD (Ding et al., 2016; Dong et al., 2016; Ma et al., 2016), and ALS (Freischmidt et al., 2013), emerge as prion-specific biomarker candidates. Other prion infected exosomal miRNAs with diagnostic potential include let-7b-5p, miR-29b-3p, miR-222-3p, and miR-342-3p; these miRNAs display inverse deregulation in prion infected exosomes (upregulation, Bellingham et al., 2012) compared to ALS (let-7b-5p in ALS patients serum, Freischmidt et al., 2013), or AD [miR-29b-ep in AD patients serum (Geekiyanage et al., 2012) and/or PBMCs (Villa et al., 2013), miR-222-3p in AD patients serum (Zeng et al., 2017) and miR-342-3p in AD serum (Tan et al., 2014)]. Other upregulated in prion infected exosomes miRNAs, such as let-7i-5p and miR-128-3p, and the downregulated miR-146a-5p, display similar deregulation patterns in MS patients exosomes (let-7i-5p, Kimura et al., 2018), Primary Progressive (PPMS) MS patients serum (miR-128-3p, Vistbakka et al., 2017) and AD (miR-146a-5p, Kiko et al., 2013; Dong et al., 2015) or PD patients serum (miR-146a-5p, Ma et al., 2016).

Further high throughput studies and subsequent validation are required to identify effective prion specific miRNA biomarkers in body fluids.

## Future Perspectives in Prion Diseases miRNA Research

To explore the functional role of dysregulated miRNA networks in prion disease pathology it is crucial to understand the contribution of specific miRNA-mRNA target interactions involved in disease mechanisms *in vivo.* Transcriptomic-wide studies, such as high-throughput sequencing of RNA isolated by RISC immunoprecipitation could allow the global identification of miRNAs and their targets in a tissue- and cell-specific manner in prion diseases. The first approach toward profiling active miRNAs in prion disease was provided by (Llorens et al., 2018), through identification of disease-relevant miRNAs bound to Argonaute proteins in sCJD brain. In order to gain a complete picture of the role of miRNA dysregulation in disease, reported miRNA alterations need to be integrated into the complex cellular context of the brain and temporal disease evolution. Temporal and cellular resolution can be achieved by single-cell RNAseq, miRNA *in situ* hybridization techniques and a broad range of experimental methods that are able to reflect *in vivo* disease processes.

miRNAs hold potential to restore dysregulated pathways within critical time periods in prion disease progression, due to their ability to simultaneously control a large number of genes (Ouellet et al., 2006). The use of artificial miRNAs targeting PrP^C^ has been demonstrated to reduce PrP^C^ expression, inhibiting prion propagation in neuroblastoma cells (Kang et al., 2011) and in primary neuronal cultures (Pfeifer et al., 2006; Kang et al., 2017). Lentivector-mediated RNAi reduction of endogenous PrP^C^ in scrapie-infected mice renders them resistant to prion infection, demonstrating the therapeutic potential of RNAi based therapy in prion diseases (Pfeifer et al., 2006; Ridolfi and Abdel-Haq, 2018). In this context, recent studies revealed exosomes as promising delivery systems, crossing the blood brain barrier (Ha et al., 2016; Chen et al., 2017; Yang et al., 2017).

Studying miRNA dysregulation in prion diseases mouse models is an appealing approach to dissect the causative involvement and consequential effects of miRNA alterations in prion disease pathology. Especially, the sCJD mouse model recapitulates disease hallmarks (Padilla et al., 2011) and resembles dysregulated gene expression networks during disease progression, including the brain region-specific miRNA alterations observed in sCJD (Llorens et al., 2014, 2018). This model provides an attractive tool to study the specific role of miRNA-regulated pathways in prion disease progression and a unique platform to assess biomarker candidates and/or therapeutic targets.

## Conclusion

We summarized the *current* knowledge on miRNA alterations and underlying cellular mechanisms in human prion diseases, highlighting potential links between impaired miRNA regulatory pathways and disease etiology. Gaining a complete picture of disease-associated miRNA signatures in a cell-type dependent manner will be the first step to new research lines in developing therapeutic strategies for prion diseases.

## Author Contributions

EK, KT, and FL conceived the idea. EK and KT drafted the review manuscript. KX, IF, DD, IZ, TS, and FL critically reviewed the article. All the authors contributed to literature review, and revised and approved the manuscript.

## Conflict of Interest Statement

The authors declare that the research was conducted in the absence of any commercial or financial relationships that could be construed as a potential conflict of interest.
